# 2-Methoxy­benzohydrazide

**DOI:** 10.1107/S1600536809038227

**Published:** 2009-09-26

**Authors:** Uzma Ashiq, Rifat Ara Jamal, Muhammad Nadeem Arshad, Zahida Tasneem Maqsood, Islam Ullah Khan

**Affiliations:** a Department of Chemistry, University of Karachi, Karachi 75270, Pakistan; b Department of Chemistry, Government College University, Lahore, Pakistan

## Abstract

The title compound, C_8_H_10_N_2_O_2_, crystallizes as two independent mol­ecules linked by N—H⋯N and N—H⋯O hydrogen bonds into a linear chain running along the *a* axis of the monoclinic unit cell. The intra- and inter­molecular hydrogen bonds are described as a two-ring *R*_2_^2^(10) motif. The six-membered *R*_1_^1^(6) rings formed by the intra­molecular inter­actions are almost planar (r.m.s. deviations 0.06 and 0.08 Å). In one mol­ecule, the aromatic and hydrogen-bonded rings are oriented at 4.8 (2)°, whereas in the other mol­ecule these rings are oriented at 6.1 (4)°.

## Related literature

For related structures, see: Ashiq *et al.* (2009 ▶); Kallel *et al.* (1992 ▶); Saraogi *et al.* (2002 ▶). For the biological activity of hydrazides, see: Ara *et al.* (2007 ▶); El-Emam *et al.* (2004 ▶); Maqsood *et al.* (2006 ▶). For graph-set notation, see: Bernstein *et al.* (1995 ▶).
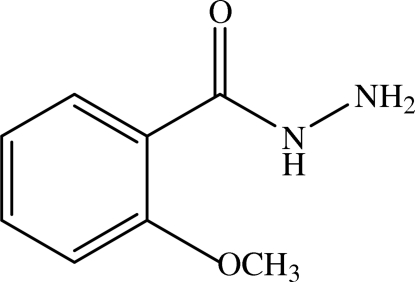


## Experimental

### 

#### Crystal data

C_8_H_10_N_2_O_2_
*M* *_r_* = 166.18
Monoclinic, 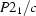

*a* = 7.6486 (5) Å
*b* = 10.7123 (7) Å
*c* = 20.4781 (13) Å
β = 95.563 (3)°
*V* = 1669.95 (19) Å^3^
*Z* = 8
Mo *K*α radiation
μ = 0.10 mm^−1^
*T* = 296 K
0.22 × 0.19 × 0.11 mm

#### Data collection

Bruker Kappa APEXII CCD diffractometer
Absorption correction: none
15129 measured reflections
2938 independent reflections
1695 reflections with *I* > 2σ(*I*)
*R*_int_ = 0.045

#### Refinement

*R*[*F* ^2^ > 2σ(*F* ^2^)] = 0.041
*wR*(*F* ^2^) = 0.110
*S* = 1.02
2938 reflections
237 parameters
H atoms treated by a mixture of independent and constrained refinement
Δρ_max_ = 0.12 e Å^−3^
Δρ_min_ = −0.16 e Å^−3^

### 

Data collection: *APEX2* (Bruker, 2007 ▶); cell refinement: *SAINT* (Bruker, 2007 ▶); data reduction: *SAINT*; program(s) used to solve structure: *SHELXS97* (Sheldrick, 2008 ▶); program(s) used to refine structure: *SHELXL97* (Sheldrick, 2008 ▶); molecular graphics: *ORTEP-3 for Windows* (Farrugia, 1997 ▶); software used to prepare material for publication: *SHELXL97*.

## Supplementary Material

Crystal structure: contains datablocks I, global. DOI: 10.1107/S1600536809038227/ng2643sup1.cif

Structure factors: contains datablocks I. DOI: 10.1107/S1600536809038227/ng2643Isup2.hkl

Additional supplementary materials:  crystallographic information; 3D view; checkCIF report

## Figures and Tables

**Table 1:** Hydrogen-bond geometry (Å, °)

*D*—H⋯*A*	*D*—H	H⋯*A*	*D*⋯*A*	*D*—H⋯*A*
N2—H21N⋯O3^i^	0.88 (2)	2.27 (2)	3.091 (3)	155 (2)
N3—H3N⋯N2^ii^	0.87 (2)	2.44 (2)	3.111 (3)	134.2 (18)
N4—H41N⋯O3^iii^	0.96 (2)	2.25 (3)	3.136 (3)	152.3 (19)
N4—H42N⋯O1^iv^	0.87 (2)	2.26 (2)	3.055 (3)	153 (2)
N1—H1N⋯O2	0.89 (2)	1.98 (2)	2.655 (2)	130.8 (17)
N3—H3N⋯O4	0.86 (2)	2.01 (2)	2.653 (2)	129.9 (19)

Symmetry codes: (i) 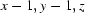; (ii) 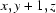; (iii) 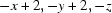; (iv) 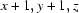.

**Fig. 1.**
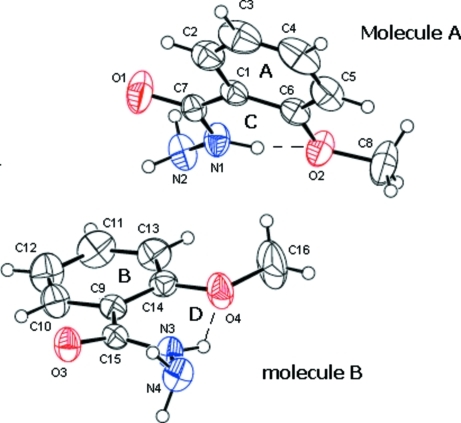
ORTEP plot of the title compound with the ellipsoids drawn at the 40% probability level, showing the atomic labels.

**Fig. 2.**
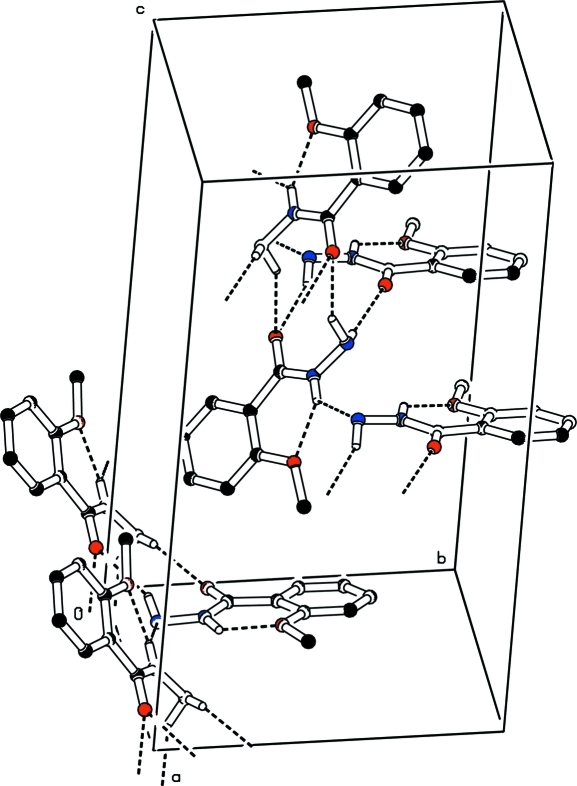
A unit cell packing diagram of (I) showing hydrogen bonds drawn by dashed lines. Hydrogen atoms not involved in H-bonding have been omitted.

## supplementary crystallographic information

### Comment

Hydrazides are known to have different biological activities and have been used for the synthesis of various heterocyclic compounds (El-Emam *et al.*, 2004). In order to study the biological activity of 2-methoxybenzohydrazide, we undertook the synthesis of title compound and report its crystal structure in this paper. The title compound I was found to be antifungal (Maqsood *et al.*, 2006) and phytotoxic (Ara *et al.*, 2007). The unit cell contains two crystallographically unique molecules (Fig. 1). The structures of benzhydrazide (Kallel *et al.*, 1992), *para*-chloro (Saraogi *et al.*, 2002) and *para*-methoxy (Ashiq *et al.*, 2009), analogues of (I) have already been reported.

The molecular packing diagram (Fig. 2) shows the presence of intermolecular hydrogen bonds of N—H···N and N—H···O types (details are given in Table 1) results in the formation of two ring motifs with graphic notation *R*_2_^2^(10) (Bernstein *et al.*, 1995), for each. Intramolecular interactions give rise six membered rings C (O2/C6/C1/C7/N1/H1N) and D (O4/C14/C9/C15/N3/H3N) *R*_1_^1^(6) (Bernstein *et al.*, 1995), in each molecule. In one molecule, the A and C rings are oriented at 4.8 (2)°, whereas in the other molecule, the B and D rings are oriented at 6.1 (4)°.

### Experimental

All reagent-grade chemicals were obtained from Aldrich and Sigma Chemical companies and were used without further purification. To a solution of ethyl-2-methoxybenzoate (3.6 g, 20 mmol) in 75 ml e thanol, hydrazine hydrate (5.0 ml, 100 mmol) was added. The mixture was refluxed for 5 h and a solid was obtained upon removal of the solvent by rotary evaporation. The resulting solid was washed with hexane to afford 2-methoxybenzohydrazide (yield 78%) (Ara *et al.*, 2007). Colourless single crystals of (I) were obtained by slow evaporation of methanol solution at room temperature.

### Refinement

The Hydrogen atoms bonded to aryl and methyl Carbon atoms were positioned geometrically, with C—H = 0.93 Å and C—H = 0.96 Å respectively. The thermal parameter of H-atoms of methyl group was taken 1.5 times of the parent C-atom, whereas for aromatic H-atoms it was taken 1.2 times of their parent atoms. Atoms H1N, H21N, H22N H3N, H41N, H42N with N–H= 0.86 (2)–0.96 (2)Å are located in a difference Fourier map and constrained to ride on their parent atom, with *U*_iso_(H) = 1.2*U*_eq_(N).

### Crystal data

**Table:**

C_8_H_10_N_2_O_2_	*F*(000) = 704
*M**_r_* = 166.18	*D*_x_ = 1.322 Mg m^−^^3^
Monoclinic, *P*2_1_/*c*	Mo *K*α radiation, λ = 0.71073 Å
Hall symbol: -P 2ybc	Cell parameters from 2389 reflections
*a* = 7.6486 (5) Å	θ = 2.7–22.7°
*b* = 10.7123 (7) Å	µ = 0.10 mm^−^^1^
*c* = 20.4781 (13) Å	*T* = 296 K
β = 95.563 (3)°	Needle, colourless
*V* = 1669.95 (19) Å^3^	0.22 × 0.19 × 0.11 mm
*Z* = 8	

### Data collection

**Table:**

Bruker Kappa APEXII CCD diffractometer	1695 reflections with *I* > 2σ(*I*)
Radiation source: fine-focus sealed tube	*R*_int_ = 0.045
graphite	θ_max_ = 25.0°, θ_min_ = 2.0°
ω scans	*h* = −9→9
15129 measured reflections	*k* = −12→12
2938 independent reflections	*l* = −24→24

### Refinement

**Table:**

Refinement on *F*^2^	Primary atom site location: structure-invariant direct methods
Least-squares matrix: full	Secondary atom site location: difference Fourier map
*R*[*F*^2^ > 2σ(*F*^2^)] = 0.041	Hydrogen site location: inferred from neighbouring sites
*wR*(*F*^2^) = 0.110	H atoms treated by a mixture of independent and constrained refinement
*S* = 1.02	*w* = 1/[σ^2^(*F*_o_^2^) + (0.0442*P*)^2^ + 0.2288*P*] where *P* = (*F*_o_^2^ + 2*F*_c_^2^)/3
2938 reflections	(Δ/σ)_max_ < 0.001
237 parameters	Δρ_max_ = 0.12 e Å^−^^3^
0 restraints	Δρ_min_ = −0.16 e Å^−^^3^

### Special details

**Table:**

Geometry. All e.s.d.'s (except the e.s.d. in the dihedral angle between two l.s. planes) are estimated using the full covariance matrix. The cell e.s.d.'s are taken into account individually in the estimation of e.s.d.'s in distances, angles and torsion angles; correlations between e.s.d.'s in cell parameters are only used when they are defined by crystal symmetry. An approximate (isotropic) treatment of cell e.s.d.'s is used for estimating e.s.d.'s involving l.s. planes.
Refinement. Refinement of *F*^2^ against ALL reflections. The weighted *R*-factor *wR* and goodness of fit *S* are based on *F*^2^, conventional *R*-factors *R* are based on *F*, with *F* set to zero for negative *F*^2^. The threshold expression of *F*^2^ > σ(*F*^2^) is used only for calculating *R*-factors(gt) *etc*. and is not relevant to the choice of reflections for refinement. *R*-factors based on *F*^2^ are statistically about twice as large as those based on *F*, and *R*- factors based on ALL data will be even larger.

### Fractional atomic coordinates and isotropic or equivalent isotropic displacement parameters (Å^2^)

**Table:**

	*x*	*y*	*z*	*U*_iso_*/*U*_eq_	
O1	0.1822 (2)	0.26307 (16)	0.04342 (10)	0.0827 (6)	
O2	0.6697 (2)	0.39866 (15)	0.11303 (8)	0.0656 (5)	
N2	0.4302 (3)	0.0831 (2)	0.06058 (12)	0.0615 (6)	
N1	0.4586 (2)	0.21149 (17)	0.07266 (9)	0.0525 (5)	
H1N	0.569 (3)	0.235 (2)	0.0811 (11)	0.063*	
H21N	0.335 (3)	0.066 (2)	0.0792 (11)	0.063*	
H22N	0.400 (3)	0.081 (2)	0.0191 (12)	0.063*	
C1	0.3759 (3)	0.43070 (19)	0.07062 (10)	0.0437 (5)	
C2	0.2404 (3)	0.5132 (2)	0.05221 (12)	0.0618 (7)	
H2	0.1317	0.4815	0.0359	0.074*	
C3	0.2614 (5)	0.6394 (3)	0.05727 (14)	0.0803 (9)	
H3	0.1683	0.6925	0.0442	0.096*	
C4	0.4197 (5)	0.6872 (3)	0.08160 (14)	0.0802 (9)	
H4	0.4341	0.7732	0.0853	0.096*	
C5	0.5589 (4)	0.6088 (2)	0.10075 (12)	0.0669 (7)	
H5	0.6664	0.6419	0.1174	0.080*	
C6	0.5379 (3)	0.4812 (2)	0.09505 (10)	0.0486 (6)	
C7	0.3323 (3)	0.2961 (2)	0.06103 (10)	0.0462 (6)	
C8	0.8411 (3)	0.4463 (3)	0.13126 (16)	0.0977 (10)	
H8A	0.8430	0.4878	0.1729	0.147*	
H8B	0.8720	0.5045	0.0986	0.147*	
H8C	0.9239	0.3787	0.1346	0.147*	
O3	1.10200 (18)	0.94743 (13)	0.10222 (7)	0.0552 (4)	
O4	0.64141 (19)	0.94752 (16)	0.19111 (7)	0.0652 (5)	
N3	0.8244 (2)	1.01543 (17)	0.09341 (9)	0.0471 (5)	
H3N	0.720 (3)	1.012 (2)	0.1063 (10)	0.057*	
N4	0.8484 (3)	1.1070 (2)	0.04533 (11)	0.0558 (5)	
H41N	0.862 (3)	1.062 (2)	0.0055 (12)	0.067*	
H42N	0.950 (3)	1.140 (2)	0.0583 (11)	0.067*	
C9	0.9165 (3)	0.85915 (19)	0.17588 (10)	0.0406 (5)	
C10	1.0450 (3)	0.7726 (2)	0.19540 (11)	0.0572 (6)	
H10	1.1445	0.7682	0.1728	0.069*	
C11	1.0305 (4)	0.6926 (2)	0.24713 (13)	0.0740 (8)	
H11	1.1178	0.6341	0.2587	0.089*	
C12	0.8864 (4)	0.7004 (3)	0.28123 (13)	0.0738 (8)	
H12	0.8768	0.6476	0.3168	0.089*	
C13	0.7564 (3)	0.7843 (2)	0.26394 (11)	0.0615 (7)	
H13	0.6591	0.7886	0.2878	0.074*	
C14	0.7681 (3)	0.8634 (2)	0.21101 (10)	0.0459 (5)	
C15	0.9534 (3)	0.94351 (18)	0.12070 (10)	0.0400 (5)	
C16	0.4932 (4)	0.9605 (4)	0.22740 (15)	0.1152 (13)	
H16A	0.4315	0.8825	0.2275	0.173*	
H16B	0.4164	1.0236	0.2075	0.173*	
H16C	0.5316	0.9842	0.2717	0.173*	

### Atomic displacement parameters (Å^2^)

**Table:**

	*U*^11^	*U*^22^	*U*^33^	*U*^12^	*U*^13^	*U*^23^
O1	0.0401 (10)	0.0727 (12)	0.1326 (16)	−0.0064 (9)	−0.0057 (10)	0.0055 (11)
O2	0.0463 (10)	0.0639 (11)	0.0838 (12)	−0.0039 (8)	−0.0079 (8)	−0.0035 (9)
N2	0.0550 (13)	0.0518 (14)	0.0805 (15)	−0.0044 (10)	0.0207 (12)	−0.0025 (12)
N1	0.0415 (11)	0.0404 (12)	0.0756 (14)	−0.0010 (10)	0.0055 (10)	−0.0032 (10)
C1	0.0458 (13)	0.0462 (14)	0.0409 (12)	0.0049 (11)	0.0136 (10)	0.0060 (10)
C2	0.0587 (15)	0.0638 (18)	0.0644 (16)	0.0143 (13)	0.0142 (12)	0.0144 (13)
C3	0.100 (2)	0.061 (2)	0.083 (2)	0.0307 (18)	0.0275 (18)	0.0185 (16)
C4	0.123 (3)	0.0454 (17)	0.079 (2)	0.0049 (19)	0.045 (2)	0.0001 (15)
C5	0.086 (2)	0.0551 (17)	0.0623 (17)	−0.0126 (15)	0.0217 (14)	−0.0100 (13)
C6	0.0554 (14)	0.0487 (15)	0.0436 (13)	0.0014 (12)	0.0136 (11)	0.0008 (11)
C7	0.0389 (13)	0.0556 (15)	0.0452 (13)	0.0005 (12)	0.0104 (10)	0.0047 (11)
C8	0.0504 (16)	0.114 (3)	0.124 (3)	−0.0211 (16)	−0.0132 (16)	−0.007 (2)
O3	0.0440 (9)	0.0614 (10)	0.0626 (10)	0.0031 (7)	0.0167 (7)	0.0124 (8)
O4	0.0523 (10)	0.0894 (13)	0.0575 (10)	0.0187 (9)	0.0243 (8)	0.0182 (9)
N3	0.0421 (11)	0.0499 (12)	0.0506 (11)	0.0010 (9)	0.0107 (9)	0.0135 (9)
N4	0.0528 (12)	0.0576 (14)	0.0571 (13)	−0.0035 (10)	0.0063 (10)	0.0184 (11)
C9	0.0446 (12)	0.0366 (12)	0.0410 (12)	−0.0033 (10)	0.0069 (10)	−0.0014 (10)
C10	0.0569 (15)	0.0503 (15)	0.0657 (16)	0.0051 (12)	0.0131 (12)	0.0071 (13)
C11	0.081 (2)	0.0605 (17)	0.0807 (19)	0.0116 (14)	0.0108 (16)	0.0273 (15)
C12	0.089 (2)	0.0649 (18)	0.0679 (18)	−0.0072 (16)	0.0074 (16)	0.0266 (15)
C13	0.0641 (17)	0.0711 (18)	0.0512 (15)	−0.0101 (14)	0.0149 (12)	0.0114 (13)
C14	0.0463 (13)	0.0507 (14)	0.0406 (13)	−0.0027 (11)	0.0039 (10)	0.0005 (11)
C15	0.0416 (12)	0.0389 (12)	0.0401 (12)	−0.0015 (10)	0.0079 (10)	−0.0031 (10)
C16	0.078 (2)	0.184 (4)	0.093 (2)	0.051 (2)	0.0534 (18)	0.039 (2)

### Geometric parameters (Å, °)

**Table:**

O1—C7	1.222 (2)	O3—C15	1.233 (2)
O2—C6	1.364 (3)	O4—C14	1.356 (2)
O2—C8	1.423 (3)	O4—C16	1.421 (3)
N2—N1	1.410 (3)	N3—C15	1.331 (3)
N2—H21N	0.88 (2)	N3—N4	1.414 (2)
N2—H22N	0.86 (2)	N3—H3N	0.87 (2)
N1—C7	1.329 (3)	N4—H41N	0.96 (2)
N1—H1N	0.88 (2)	N4—H42N	0.87 (2)
C1—C2	1.386 (3)	C9—C10	1.382 (3)
C1—C6	1.399 (3)	C9—C14	1.403 (3)
C1—C7	1.488 (3)	C9—C15	1.495 (3)
C2—C3	1.364 (4)	C10—C11	1.375 (3)
C2—H2	0.9300	C10—H10	0.9300
C3—C4	1.364 (4)	C11—C12	1.363 (3)
C3—H3	0.9300	C11—H11	0.9300
C4—C5	1.383 (4)	C12—C13	1.362 (3)
C4—H4	0.9300	C12—H12	0.9300
C5—C6	1.380 (3)	C13—C14	1.385 (3)
C5—H5	0.9300	C13—H13	0.9300
C8—H8A	0.9600	C16—H16A	0.9600
C8—H8B	0.9600	C16—H16B	0.9600
C8—H8C	0.9600	C16—H16C	0.9600
			
C6—O2—C8	118.5 (2)	C14—O4—C16	119.39 (19)
N1—N2—H21N	104.3 (15)	C15—N3—N4	123.54 (18)
N1—N2—H22N	102.9 (16)	C15—N3—H3N	120.8 (14)
H21N—N2—H22N	105 (2)	N4—N3—H3N	115.5 (15)
C7—N1—N2	122.49 (19)	N3—N4—H41N	105.8 (14)
C7—N1—H1N	120.3 (15)	N3—N4—H42N	104.1 (15)
N2—N1—H1N	116.3 (15)	H41N—N4—H42N	107 (2)
C2—C1—C6	117.6 (2)	C10—C9—C14	117.5 (2)
C2—C1—C7	115.5 (2)	C10—C9—C15	116.33 (18)
C6—C1—C7	126.91 (19)	C14—C9—C15	126.12 (19)
C3—C2—C1	122.1 (3)	C11—C10—C9	122.2 (2)
C3—C2—H2	118.9	C11—C10—H10	118.9
C1—C2—H2	118.9	C9—C10—H10	118.9
C2—C3—C4	119.6 (3)	C12—C11—C10	119.1 (2)
C2—C3—H3	120.2	C12—C11—H11	120.4
C4—C3—H3	120.2	C10—C11—H11	120.4
C3—C4—C5	120.4 (3)	C13—C12—C11	120.9 (2)
C3—C4—H4	119.8	C13—C12—H12	119.6
C5—C4—H4	119.8	C11—C12—H12	119.6
C6—C5—C4	119.9 (3)	C12—C13—C14	120.4 (2)
C6—C5—H5	120.1	C12—C13—H13	119.8
C4—C5—H5	120.1	C14—C13—H13	119.8
O2—C6—C5	122.8 (2)	O4—C14—C13	122.9 (2)
O2—C6—C1	116.9 (2)	O4—C14—C9	117.24 (18)
C5—C6—C1	120.3 (2)	C13—C14—C9	119.9 (2)
O1—C7—N1	120.0 (2)	O3—C15—N3	121.28 (19)
O1—C7—C1	120.8 (2)	O3—C15—C9	119.98 (19)
N1—C7—C1	119.20 (19)	N3—C15—C9	118.72 (17)
O2—C8—H8A	109.5	O4—C16—H16A	109.5
O2—C8—H8B	109.5	O4—C16—H16B	109.5
H8A—C8—H8B	109.5	H16A—C16—H16B	109.5
O2—C8—H8C	109.5	O4—C16—H16C	109.5
H8A—C8—H8C	109.5	H16A—C16—H16C	109.5
H8B—C8—H8C	109.5	H16B—C16—H16C	109.5
			
C6—C1—C2—C3	0.1 (3)	C14—C9—C10—C11	0.0 (3)
C7—C1—C2—C3	−179.1 (2)	C15—C9—C10—C11	177.5 (2)
C1—C2—C3—C4	−0.5 (4)	C9—C10—C11—C12	−1.3 (4)
C2—C3—C4—C5	0.4 (4)	C10—C11—C12—C13	1.2 (4)
C3—C4—C5—C6	0.2 (4)	C11—C12—C13—C14	0.2 (4)
C8—O2—C6—C5	−7.4 (3)	C16—O4—C14—C13	3.1 (3)
C8—O2—C6—C1	173.2 (2)	C16—O4—C14—C9	−176.4 (2)
C4—C5—C6—O2	−180.0 (2)	C12—C13—C14—O4	179.1 (2)
C4—C5—C6—C1	−0.7 (3)	C12—C13—C14—C9	−1.5 (3)
C2—C1—C6—O2	179.89 (18)	C10—C9—C14—O4	−179.18 (19)
C7—C1—C6—O2	−1.1 (3)	C15—C9—C14—O4	3.7 (3)
C2—C1—C6—C5	0.5 (3)	C10—C9—C14—C13	1.3 (3)
C7—C1—C6—C5	179.5 (2)	C15—C9—C14—C13	−175.8 (2)
N2—N1—C7—O1	5.1 (3)	N4—N3—C15—O3	−4.7 (3)
N2—N1—C7—C1	−175.71 (19)	N4—N3—C15—C9	173.79 (19)
C2—C1—C7—O1	−6.0 (3)	C10—C9—C15—O3	−11.5 (3)
C6—C1—C7—O1	175.0 (2)	C14—C9—C15—O3	165.7 (2)
C2—C1—C7—N1	174.84 (19)	C10—C9—C15—N3	169.98 (19)
C6—C1—C7—N1	−4.2 (3)	C14—C9—C15—N3	−12.8 (3)

### Hydrogen-bond geometry (Å, °)

**Table:**

*D*—H···*A*	*D*—H	H···*A*	*D*···*A*	*D*—H···*A*
N2—H21N···O3^i^	0.88 (2)	2.27 (2)	3.091 (3)	155 (2)
N3—H3N···N2^ii^	0.87 (2)	2.44 (2)	3.111 (3)	134.2 (18)
N4—H41N···O3^iii^	0.96 (2)	2.25 (3)	3.136 (3)	152.3 (19)
N4—H42N···O1^iv^	0.87 (2)	2.26 (2)	3.055 (3)	153 (2)
N1—H1N···O2	0.89 (2)	1.98 (2)	2.655 (2)	130.8 (17)
N3—H3N···O4	0.86 (2)	2.01 (2)	2.653 (2)	129.9 (19)

Symmetry codes: (i) *x*−1, *y*−1, *z*; (ii) *x*, *y*+1, *z*; (iii) −*x*+2, −*y*+2, −*z*; (iv) *x*+1, *y*+1, *z*.
